# Early life exposure to ambient particulate matter and Kawasaki disease: a systematic review and meta-analysis

**DOI:** 10.3389/fcvm.2025.1611757

**Published:** 2025-08-11

**Authors:** Christina Moncur, Mona Kamotho, Tanisha Jain, Noah Weslock, Mark Ragheb, Kerry Mitchell

**Affiliations:** Department of Public Health & Preventive Medicine, School of Medicine, St. George’s University, True Blue, Grenada

**Keywords:** Kawasaki disease, particulate matter, vascular disease, inflammation, PM_10_, PM_2.5_

## Abstract

**Introduction:**

Though numerous air pollutants have been mechanistically associated with inflammation and vascular morbidity, particulate matter (PM) is one of the pollutants with the strongest association. However, PM is generally categorized according to aerodynamic diameters between 2.5 and 10 μg m^−3^ (PM_10_) and less than 2.5 μg m^−3^ (PM_2.5_). Given their differential ability to enter the bloodstream, these sizes play a crucial role in the local or systemic inflammatory responses elicited after exposure. Given that vascular inflammation is a key marker of Kawasaki Disease (KD), this systematic review aims to summarize the available data on the association between KD and perinatal and early childhood PM exposure and identify any pathophysiological links.

**Methods:**

A systematic search was conducted using PubMed and EMBASE. Studies with PM included as a predictor, and Kawasaki disease as an outcome were included. This review followed the Preferred Reporting Items for Systematic Reviews and Meta-Analyses guidelines. The risk of bias in the selected studies was assessed using the QUADAS-2 tool.

**Results:**

Eleven studies met the criteria for inclusion. All studies suggested an association between exposure to PM and increase risk or exacerbation of KD, though not all results reached statistical significance. Due to significant heterogeneity, pooled analyses were possible only in select studies for pre- and postnatal PM_10_ exposure and postnatal PM_2.5_ exposure. All studies identified immune-mediated inflammatory responses as a key pathophysiological link between exposure and KD, with PM_10_ noted as a significant risk factor for respiratory inflammation and poor maternal and child health, and PM_2.5_ for a wide range of adverse outcomes, especially in children and populations with preexisting inflammatory diseases. The role social, behavioral and environmental modifiers play in disease incidence was also highlighted.

**Conclusion:**

Particulate matter exposure is associated with an increased risk of developing and exacerbating KD, especially in populations experiencing temporary increased sensitivity and in populations with preexisting inflammatory diseases.

**Systematic Review Registration:**

https://www.crd.york.ac.uk/PROSPERO/view/CRD42023468937, PROSPERO CRD42023468937.

## 1 Introduction

Air pollution is associated with significant global morbidity and mortality, with 2021 estimates ranking it as the second leading risk factor for early death, contributing to 8.1 million deaths (1). Mechanistic studies have demonstrated the causative effect of exposure to numerous air pollutants. However, particulate matter, with aerodynamic diameters ranging from between 2.5 and 10 μg m^−3^ (PM_10_) and less than 2.5 μg m^−3^ (PM_2.5_) is one of the pollutants most strongly associated with adverse health effects. PM_2.5_ has notably demonstrated an ability to cross into the circulatory system, causing systemic toxic effects, including but not limited to respiratory, neurological, and cardiovascular dysfunction, typically driven by inflammatory and immunological responses (2–4). This systemic dysfunction is closely linked to higher rates of asthma, chronic lung disease, diabetes, cognitive decline, and various cardiovascular diseases (5–8). The impact of particulate matter exposures on the cardiovascular system is significant, given the global morbidity associated with cardiovascular diseases (9). Notably, cardiovascular diseases currently represent the top three leading causes of death, while air pollution ranks as the second leading risk factor (1, 9). Studies have demonstrated that exposure to PM, especially PM_2.5_, in pediatric populations is linked to an increase the risk of adverse cardiovascular outcomes, both in the short and long term (3, 10). Although various biological mechanisms play a role in these outcomes, they are generally the cumulative result of oxidative stress and endothelial dysfunction caused by vascular inflammation (8, 11).

Kawasaki disease (KD) is an example of an inflammatory vascular disease predominantly affecting pediatric populations under the age of five. It is characterized by inflammation of medium-sized arteries, especially the coronary arteries, and can lead to serious cardiovascular complications, including myocardial infarction, if not promptly treated (12). Although the etiology remains unclear, KD is believed to result from a dysregulated immune response triggered by environmental or infectious agents in genetically susceptible individuals (13). The burden of KD varies significantly across different countries, with the highest incidence found in North-East Asian countries. For example, in Japan, almost 1 in 100 children are affected by the disease by the age of five. In contrast, the lowest incidence is reported in sub-Saharan Africa (14). Globally, the incidence of KD has been increasing, particularly in East Asian countries and may partly be due to increased diagnostic awareness and environmental changes including increased prevalence of air pollutants (15, 16). Given that KD is characterized by inflammatory and immunological responses, the link between exposure to air pollutants, especially those that are causally linked to inflammatory outcomes, and the risk of development and exacerbation of KD is becoming clear (17–19). This justifies the focus on particulate matter, given its role as a trigger for immune-mediated inflammation in genetically susceptible children, which may lead to the onset or exacerbation of KD symptoms (20).

Thus, the objectives of this review are to: (1) summarize the available evidence on the association between perinatal and early childhood exposure to particulate matter and the risk and severity of Kawasaki disease, and (2) describe the likely pathophysiological connection between the disease and the pollutant in its varying aerodynamic diameters. Results from this review will add to the body of evidence on the influence of air pollution on the development and progression of Kawasaki disease, thus increasing indices of suspicion of these exposures during diagnosis and treatment.

## 2 Methods

### 2.1 Data sources and search strategy

A comprehensive search on PubMed and EMBASE was used to identify relevant studies, using the keywords (kawasaki) AND/OR (kawasaki disease) AND/OR mucocutaneous lymph node syndrome AND (particulate matter) AND/OR air pollution (Supplementary S1). Due to limited studies on this specific disease outcome, no date restrictions were applied to ensure all relevant studies were included.

### 2.2 Study selection and eligibility

This systematic review followed the guidelines of the Preferred Reporting Items for Systematic Reviews and Meta-Analyses (PRISMA). Citations retrieved from each database were imported into the reference manager Zotero v7.0.11, and duplicate entries were eliminated. Title and abstract screening by two independent reviewers (MK and TJ) determined article eligibility for inclusion. Subsequently, full texts of articles meeting established criteria were further assessed for relevance to this review. Discrepancies in eligibility assessments were resolved through discussion and arbitration by a third reviewer (CM). Studies were deemed eligible for inclusion in the review if they adhered to the following criteria: (1) employed a cohort, case-control, or cross-sectional design; (2) investigated or included the analysis of the prevalence or incidence of Kawasaki disease as an outcome, exposure to particulate matter as a predictor; and (3) incorporated a reference group. Excluded were (1) publications based on cell or animal model data and (2) reviews, commentaries, abstracts, and case reports (Supplementary S2). The protocol for this systematic review was registered with the International Prospective Register of Systematic Reviews (PROSPERO) with registration number CRD42023468937.

### 2.3 Data extraction and quality assessment

Two independent reviewers (MK and TJ) extracted the following information studies that met eligibility criteria: author, study site, design and sample size, objectives, and key findings. Disputes during bias assessments were resolved through discussion and arbitration by a third reviewer (CM). The risk of bias in selected studies was evaluated using the QUADAS-2 tool by NW and MR (Supplementary S3).

### 2.4 Statistical analysis

Statistical analysis was conducted to compare mean differences in PM_10_ and PM_2.5_ levels between individuals with Kawasaki disease and controls. Given the low heterogeneity among studies as indicated by the *I*^2^ statistic, fixed-effects (common-effects) meta-analyses were performed only for prenatal and postnatal PM_10_ levels, and postnatal PM_2.5_ levels. The mean difference (MD) was used as the summary measure, with 95% confidence intervals (CIs) calculated for each estimate. Forest plots were generated to visually summarize the meta-analysis results, showing individual study estimates with their weights, as well as the pooled MD and 95% CI. Analyses were conducted using the *meta* package in R, with the *metacont* function used to compute pooled effect estimates. Statistical significance was evaluated at *p* < 0.05. Publication bias was not assessed due to the small number of studies (*n* = 2). All statistical analyses were conducted using RStudio 2025.05.0 Build 496 (21).

## 3 Results

### 3.1 Study selection and quality assessment

**Figure 1:**
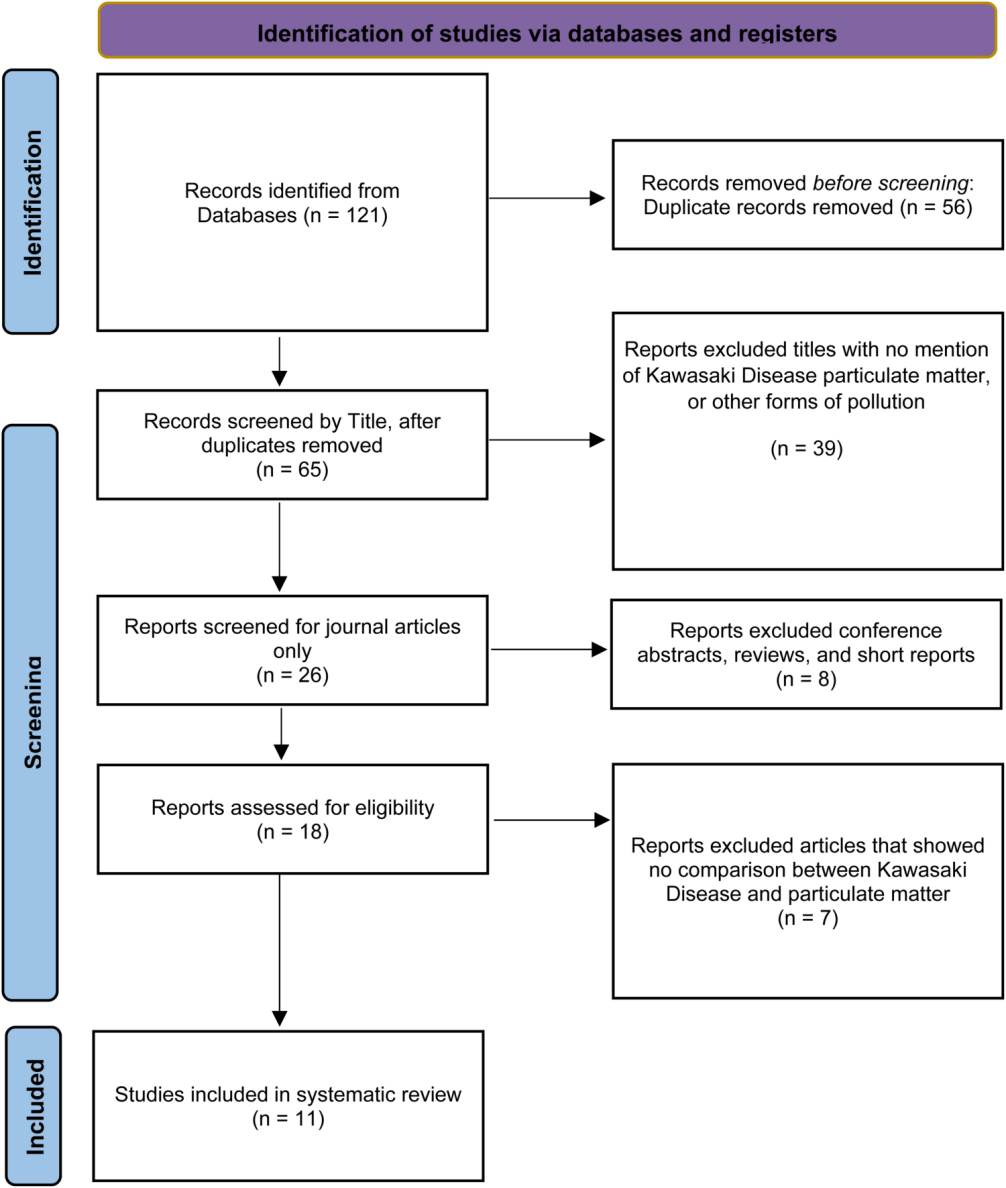
Flow diagram of the study selection process using the preferred reporting items for systematic reviews and meta-analyses.

A total of 121 papers met the initial search criteria. After removing duplicates and conducting an initial eligibility screening, 18 papers were selected for full-text review. This review resulted in a final selection of 11 papers that met the full eligibility criteria (Figure 1). These criteria included the use of observational study designs to quantitatively assess exposure to particulate matter (PM_10_ and PM_2.5_), regardless of source; measurable changes in risk, incidence, or prevalence of Kawasaki disease in prenatal and pediatric populations; and inclusion of a reference group (Figure 1).

The methodological quality of the selected studies was assessed using the QUADAS-2 tool. Despite a generally low risk of bias across the domains of subject selection, reference standard, and flow and timing, a high risk of bias was observed in the index test domain. This is partly due to the inherent limitations of environmental exposure studies, which rely on local or regional ambient particulate matter concentrations rather than a specific diagnostic test. Additionally, the selected studies did not specify a threshold but instead used *post hoc* statistical modeling (Supplementary S3).

### 3.2 Summary analysis of selected studies

A summary of the results is presented in Table 1. Although results varied across studies, positive associations were reported between exposure to particulate matter and increased KD risk. However, only cohort and case-control studies on PM_2.5_ showed statistically significant associations. These studies typically involved large sample sizes and longitudinal exposure assessments. In contrast, case-crossover studies assessing short-term exposure showed mixed results, despite similar directional trends.

**Table 1:** Characteristics of eleven included studies on the association between PM exposure and Kawasaki disease.

Reference	Country affected	Study design	Sample number	Results	Findings
Buteau et al., (36)	Canada	Retrospective cohort Predictor: prenatal exposure; mean annual PM_2.5_ concentrations obtained from satellite estimates and regression models Outcome: KD incidence in children	505,336 children, including 539 with KD	Ambient PM_2.5_ exposure associated with KD incidence: HR = 1.16 (95%CI: 0.96–1.39). Industrial PM_2.5_ exposure associated with KD: HR = 1.01 (95%CI: 0.97–1.05).	Observed association between PM_2.5_ and KD incidence. Findings not statistically significant. PM_10_ was not studied.
Jung et al., (31)	Taiwan	Case-crossover (time stratified) Predictor: hourly PM_10_ concentrations from fixed sites Outcome: Hospitalization of children with KD	695 KD hospital admissions	IQR (40.60 μg m^−3^) increase in PM_10_ concentration associated with KD hospitalization: aOR 1.10 (95%CI: 0.94–1.30).	Observed association between PM_10_ and KD hospitalizations. Findings not statistically significant. PM_2.5_ was not studied.
Kim et al., (37)	South Korea	Cohort Predictor: monthly PM_2.5_ concentrations from an ensemble model Outcome: KD incidence in children	134,634 individuals, including 1,220 that developed KD	Annual 5 μg m^−3^ increase in PM_2.5_ concentration associated with KD incidence: HR = 1.21 (95%CI: 1.05–1.39).	Observed association between PM_2.5_ and KD incidence. Location, income, sex, and age were important predictors of the HR of PM_2.5_ on KD. Findings statistically significant.
Kuo et al., (24)	Taiwan	Case-control Predictor: pre-and postnatal exposure; monthly composite pollutant standards index (PSI) concentrations from fixed sites Outcome: KD incidence in children	16,768 without KD, 4,192 with KD	Prenatal exposure to PSI and KD incidence: OR = 1.01 (95%CI; 1.00–1.01). Postnatal exposure to PSI and KD incidence after pregnancy: 1.00 (95%CI; 1.00–1.01).	PSI included SO_2_, NO_2_, O_3_, CO, and PM_10_ were positively associated with KD incidence before and after pregnancy. Findings are borderline statistically significant.
Kwon et al., (30)	South Korea	Case-crossover (time stratified) Predictor: daily PM_2.5_ concentrations from fixed sites Outcome: KD incidence in children	51,486 children that were treated for KD	At lag 0, IQR (14.67 μg m^−3^) increase PM_2.5_ concentration associated with KD incidence: aOR = 1.007 (95%CI: 0.994–1.020). At lag 0, IQR (28.68 μg m^−3^) increase PM_10_ concentration associated with KD incidence: aOR = 1.003 (95%CI: 0.992–1.015). At lag 1, IQR (14.67 μg m^−3^) increase PM_2.5_ concentration associated with KD incidence: aOR = 1.016 (95%CI: 1.004–1.029). At lag 0, IQR (28.68 μg m^−3^) increase PM_10_ concentration associated with KD incidence: aOR = 1.010 (95%CI: 0.999–1.022).	Observed association between PM_10_, PM_2.5_ and KD risk and lag 0 and lag 1. Only the findings for PM_2.5_ at lag 1 and KD risk were statistically significant. Lag 0 = day of fever onset Lag 1 = day preceding fever onset
Lin et al., (29)	China	Cohort Predictor: daily PM_10_ concentrations from fixed sites Outcome: KD incidence in children	2,344 KD cases	At lag 0, PM_10_ exposure associated with percent increase in KD: 0.04% (95% CI: −1.34%, 1.34%) At lag 1, PM_10_ exposure associated with percent increase in KD: 1.05% (95% CI: −0.31%, 2.43%) At lag 2, PM_10_ exposure associated with percent increase in KD: 1.48% (95% CI: −1.07%, 1.89%)	Consistently positive associations between PM_10_ and daily Kawasaki disease (KD) incidence. Findings not statistically significant. PM_2.5_ was not studied. Lag 0 = current day of hospitalization Lag 1 = day preceding hospitalization Lag 2 = two days preceding hospitalization
Oh et al., (25)	South Korea	Case-crossover Predictor: atmospheric reanalysis Outcome: Children hospitalizations for KD	771 KD cases	At lag 0–2, PM_2.5_ and KD hospitalization: OR = 1.00 (95% CI: 0.95, 1.06). At lag 7, PM_2.5_ and KD hospitalization: OR = 0.99 (95% CI: 0.91, 1.07). At lag 14, PM_2.5_ and KD hospitalization: OR = 0.93 (95% CI: 0.82, 1.05). Subgroup analyses (adjusting for multiple predictor pollutants): At lag 0–2, PM_2.5_ and KD hospitalization: OR = 1.01 (95% CI: 0.96, 1.06); adjusted for SO_2_. At lag 0–2, PM_2.5_ and KD hospitalization: OR = 1.03 (95% CI: 0.98, 1.10); adjusted for NO_2_. At lag 0–2, PM_2.5_ and KD hospitalization: OR = 1.02 (95% CI: 0.95, 1.09); adjusted for CO. At lag 0–2, PM_2.5_ and KD hospitalization: OR = 1.01 (95% CI: 0.96, 1.07); adjusted for O_3_.	No statistically significant association was found between KD and short-term PM_2.5_ exposure. PM_10_ was not studied in multi-pollutant models. Lag 0 -2 = two day moving average Lag 7 = seven day moving average Lag 14 = fourteen day moving average
Si et al., (19)	China	Case-crossover Predictor: monthly mean PM concentrations from fixed sites Outcome: KD incidence in children	3,036 KD cases	Each 1 μg m^−3^ increase in PM_2.5_ concentration associated with increase in KD incidence: OR 0.22 (95% CI: 0.01, 0.42). Each 1 μg m^−3^ increase in PM_10_ concentration associated with decrease in KD incidence: OR −0.23 (95% CI: −0.67, 0.21).	Observed positive association between PM_2.5_ and KD incidence. Findings statistically significant. Observed negative association between PM_10_ and KD incidence. Findings not statistically significant. Statistical potentially limited due to use of seasonal statistics instead of daily statistics.
Yoneda et al., (17)	Japan	Retrospective cohort Predictor: monthly and annual PM concentration from fixed sites Outcome: KD incidence	55,289 KD cases before COVID and 14,023 KD cases after	Before COVID-19 pandemic, each 1 μg m^−3^ annual PM_2.5_ exposure associated with KD incidence rate ratio: IRR = 1.03 (95%CI: 1.01–1.06) During COVID-19 pandemic, each 1 μg m^−3^ annual PM_2.5_ exposure associated with KD incidence rate ratio: IRR = 1.10 (95%CI: 1.04–1.17)	Observed link between annual exposure to PM_2.5_ and the onset of KD. Findings statistically significant.
Yorifuji et al., (23)	Japan	Cohort Predictor: pre-and postnatal; monthly SPM concentrations from fixed sites Outcome: Children hospitalizations for KD	22,358 children, including 193 KD hospital admissions	Suspended particulate matter (SPM) associated with KD hospitalization: Prenatal Exposure to SPM > 25 μg/m^3^ vs. less than 20 μg m^−3^ with an OR = 1.59 (95%CI: 1.06–2.38) Postnatal exposure to SPM > 25 μg/m^3^ vs. less than 20 μg m^−3^ with an OR was 1.41 (95%CI: 0.82–2.41).	Stronger link between prenatal exposure and KD hospitalization compared to postnatal exposure.
Zeft et al., (47)	US and Canada	Case-crossover Predictor: daily PM_2.5_ concentration from fixed sites Outcome: KD onset in children	3,009 KD cases	The OR for a 2-day moving average for KD association with an increase of 10 μg m^−3^ in PM_2.5_ exposure was 0.980 (95%CI: 0.915–1.050).	No evidence of an association between PM_2.5_ and KD was found when studying a 2-day moving average.

Note: Studies arranged in alphabetical order.

### 3.3 Pooled analysis of selected studies

Due to significant methodological heterogeneity, a meta-analysis of all studies was not appropriate. This heterogeneity stemmed partly from differing outcome measures and regression results. Outcomes were assessed in various ways, with some studies using data from fixed-site monitoring stations and others relying on satellite-based estimations. Predictor variables also varied significantly, with some studies evaluating short-term exposure and others focusing on long-term exposure, leading to notable incomparability when discussing biological mechanisms. Finally, one study notably assessed exposure to suspended particulate matter (SPM) which corresponds to all particles with aerodynamic diameters < 10 µm, with a 50% cut-off at 7 µm, functionally categorizing it as PM_10_ (22). Nevertheless, comparisons with PM_10_ should acknowledge this source variability. However, meta-analyses were possible using subset data for pre- and postnatal PM_10_ exposure (23, 24) and postnatal PM_2.5_ exposure (24, 25) as they met criteria and provided data to estimate the mean difference in PM exposure between individuals with Kawasaki and controls.

**Figure 2:**
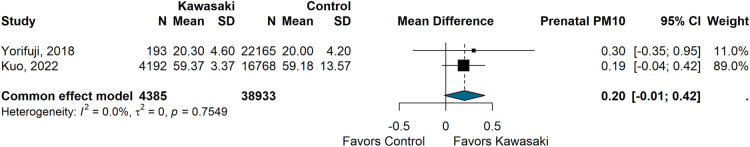
Pooled mean difference in prenatal PM_10_ exposure and children with Kawasaki disease and controls.

**Figure 3:**
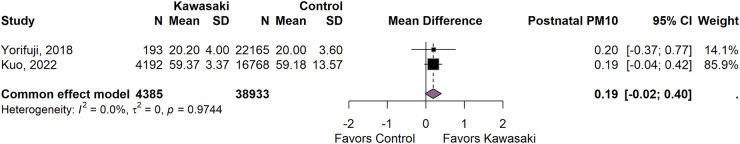
Pooled mean difference in postnatal PM_10_ exposure and children with Kawasaki disease and controls.

**Figure 4:**
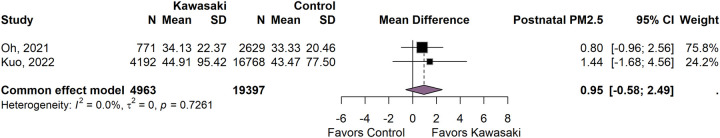
Pooled mean difference in postnatal PM_2.5_ exposure and children with Kawasaki disease and controls.

The pooled analysis of prenatal PM_10_ exposure levels among children diagnosed with Kawasaki disease compared to controls, based on two eligible studies (*n* = 43,318), is shown in Figure 2. The pooled mean difference in PM_10_ concentration was 0.20 µg m^−3^ (95% CI: −0.01 to 0.42), indicating a modest elevation in prenatal exposure among KD cases. Heterogeneity was absent (*I*^2^ = 0, *τ*^2^ = 0), suggesting high consistency between studies. However, the result did not reach statistical significance. Similarly, the pooled analysis of postnatal PM_10_ exposure in Figure 3 shows a mean difference of 0.19 µg m^−3^ (95% CI: −0.04 to 0.46) between children diagnosed with Kawasaki disease and controls (*n* = 43,318). Heterogeneity was also absent, indicating consistent results. However, the findings were not statistically significant, suggesting that within the 0–10 µg/m^3^ exposure range, postnatal ambient PM_10_ levels do not differ meaningfully between cases and controls. Finally, the pooled analysis of two studies evaluating postnatal PM_2.5_ exposure between Kawasaki disease cases and controls (*n* = 24,360) also found no statistically significant difference (Figure 4). The pooled mean difference was 0.95 µg/m^3^ (95% CI: −0.58 to 2.49). While not statistically significant, the point estimate suggests higher exposure among KD cases.

## 4 Discussion

### 4.1 PM_10_ and KD

Inhalation of PM_10_ particles leads to the penetration of the upper respiratory system and lungs, triggering an inflammatory cascade through oxidative stress and the activation of alveolar macrophages and dendritic cells (26). This activation results in the release of several pro-inflammatory cytokines, including IL-6, IL-1β, and TNF-α which promotes systemic inflammation and oxidative stress (27). Following penetration of the upper respiratory tract, PM_10_ disrupts tight junctions between endothelial cells leading to increased vascular permeability and in turn may be associated with the vascular inflammation characteristic of KD (28). Despite the biological plausibility, the epidemiological studies included in this review have yielded inconsistent findings on the association between PM_10_ and KD.

The nationwide longitudinal study in Japan performed by Yorifuji et al. on prenatal and postnatal exposure to suspended particulate matter (SPM) provided supporting evidence for the potential role of PM_10_ in the development of KD (23). Notably, the ORs for SPM exposure ≥25 µg m^−3^ compared with <20 µg m^−3^ was 1.59 (95% CI 1.06, 2.38). The authors also noted that SPM exposure during mid-to-late gestation appeared to significantly increase the risk of developing KD (23). Despite not reaching statistical significance, the meta-analysis suggests that prenatal exposure to PM_10_ increase susceptibility of exposed children to KD (23, 24), likely due to its ability to cross the placenta and alter immune system development (Figure 2). Postnatal exposure was also associated with a slight increase in KD risk (Figure 3). Lin et al. in Shanghai, China and Kwon et al. in Korea also showed an increase in KD incidence following exposure to PM_10_, also did not reach statistical significance (29, 30). Other studies that utilized population data from Taiwan by Jung et al. and Kuo et al. found no significant association between PM_10_ and KD entirely (24, 31). This suggests that while PM_10_ may contribute to KD pathogenesis, its impact may be weaker than that of other air pollutants and particulate matter with smaller aerodynamic diameters.

One potential explanation for the weak association between PM_10_ and KD is the larger size of the particles in comparison to finer particulate matter (PM_2.5_) and other air pollutants, which might limit their ability to penetrate deep in the alveolar system and get into systemic circulation (32). As a result, PM_10_ is generally associated with localized respiratory inflammation rather than the systematic immune response required to induce KD (33). Additionally, variations in study design may have contributed to the weaker association between PM_10_ and KD. Another crucial limitation across all studies is the lack of genetic stratification, despite KD's well-established link to genetic predisposition. Genetic factors, such as polymorphisms in the CXCL10/IP10 gene, which encodes a chemokine involved in immune function, have been linked to an increased risk of KD, supporting a causal relationship with susceptibility to environmental triggers such as PM_10_ (34, 35). This suggests that genetic susceptibility may act as a significant modifier, and if not accounted for, studies may underestimate the impact of PM_10_ on susceptible children.

### 4.2 PM_2.5_ and KD

PM_2.5_ is characterized by its ability to penetrate the alveoli and enter the bloodstream, triggering a significant inflammatory response (3, 4). In pooled analyses, postnatal exposure is associated with a slight but not statistically significant increase in KD risk (Figure 4). Despite the debate surrounding the precise etiology of KD, the associated vasculitis is known to involve inflammatory and immune responses, supporting the hypothesis that PM_2.5_ may be a significant environmental trigger (36). Buteau et al. found a positive association between ambient PM_2.5_ and KD at a Hazard Ratio for interquartile range increments of air pollutant exposure (HR_IQR_) of 1.16. Despite not reaching statistical significance, the results also suggested a greater susceptibility of those with preexisting diabetes for KD with respect to the increased inflammatory processes (37). Kim et al. corroborated these findings but with a statistically significant HR of 1.21 for each 5 μg/m^3^ increase in the annual PM_2.5_ concentration and a greater correlation when considering subpopulations (37). They specifically looked at biological sex and found that biological females could be affected more readily by oxidative stress, strengthening the idea that PM_2.5_ induces an immune response, which in turn induces KD. Their subgroup analysis showed that for biological females, the HR was 1.39 (95% CI: 1.12–1.73) for each 5 μg/m^3^ increase in PM_2.5_ concentration, while for biological males, the HR was 1.09 (95% CI: 0.90–1.31) (38). This may be partly due to the higher susceptibility of biological females to oxidative stress and greater immune response following exposure to air pollution (39). Additionally, biological females may be more sensitive to air pollutants in early life and more prone to inflammatory responses that could trigger the onset of KD (30).

Kwon et al. found a statistically significant effect of PM_2.5_ on KD at lag 1 (one day before the onset of KD), suggesting a possible short-term impact consistent with an immunological trigger (30, 37). Nevertheless, the statistical significance of the daily mean results observed by Kwon et al. and the annual mean results observed by Kim et al. suggest that while short-term PM_2.5_ exposure results in an acute inflammatory response, chronic exposure could also lead to prolonged inflammation and immune dysfunction (25). Oh et al. 2021, observed a similar outcome to Kwon et al., with the 2-day average of PM_2.5_ concentration showing an OR of 1.01 though no positive associations with the 7-day or 14-day averages (23, 25, 36). Si et al. and Yoneda et al. also found statistically significant increases in KD incidence during high vs. low PM_2.5_ seasons, with Yoneda et al. observing a consistent 3%–10% rise in KD incidence for each 1 μg/m^3^ increase in annual PM_2.5_ exposure (17, 19, 37).

Despite variations in statistical significance, all reviewed studies discussed systemic inflammation and immune dysregulation as the primary mechanisms involved in the pathogenesis of KD as a result of both direct inhalation of particulate matter and epigenetic effects. The authors suggest that inhalation of particulate matter is associated with changes in adaptive immune responses and induces cytokine-mediated endothelial cell damage, particularly in genetically predisposed populations (29). A link has also been suggested between perinatal exposure to air pollutants and maternal systemic inflammation, placental inflammatory disorders, and altered fetal immune responses, all of which are implicated in the disease's development. Although statistical significance was not achieved in subgroup analyses of PM exposure alone, findings from multipollutant models highlight the importance of evaluating both synergistic interactions and cumulative exposures when assessing risk (23).

### 4.3 Social, behavioral and environmental modifiers

In addition to evaluating multipollutant scenarios, it is also important to consider the social and environmental modifiers of KD incidence. Lin et al. evaluated the impact of high temperatures, described as the 99th percentile (32.4°C), and found a statistically significant increase in KD incidence with a cumulative relative risk of 1.91 (95% CI: 1.13, 3.23) compared to 10°C (37). On the other hand, Yorifuji et al. found that cold weather was also associated with an increased risk of hospitalization, with an odds ratio of 1.51 (95% CI: 1.07, 2.12) for a 20–25 µg/m^3^ exposure (36). The observed reduction in KD incidence during the COVID-19 pandemic years supports the role behavioral modifiers play in disease incidence. This decline coincided with decreased PM_2.5_ levels and the widespread implementation of non-pharmaceutical interventions, including mask mandates and social distancing measures, which ultimately reduced exposure to air pollutants, including particulate matter (37). A similar behavioral influence was observed when adjusting for maternal smoking during pregnancy. Buteau et al. reported a higher incidence rate among children of smoking mothers (19.8 cases per 100,000 person-years) compared to those of non-smoking mothers (12.3), again highlighting the role behavior can play in exposure levels and disease incidence (36). Socioeconomic modifiers also play a significant role in disease incidence. Populations from lower income settings were found to have higher KD incidence likely due to the cumulative burden of inadequate housing and healthcare access, as well as a limited ability to reduce exposure through solutions such as air purifiers (40). Buteau et al. specifically adjusted for neighborhood socioeconomic status and found higher incidence rates in areas of high material deprivation, at 13.4 cases per 100,000 person-years, compared to between 10.1 and 12.9 cases in areas with lower levels of material deprivation (41). These findings emphasize the importance of incorporating social, behavioral and environmental modifiers in future exposure assessment models, thus strengthening risk stratification and intervention targeting.

### 4.4 Study limitations and future perspectives

Although the studies in this review reported an overall positive association between particulate matter exposure and KD risk, several methodological limitations may affect the reliability of these findings. Firstly, the total number of reviewed studies was small, which highlights the need for additional primary research. Secondly, many studies relied on environmental exposure measurements, such as fixed-site or satellite observations, rather than individual-level assessments which may not accurately reflect personal exposure levels (42). Environmental data carries notable causal uncertainty, as generalized exposure measurements may underestimate the true effects (43). These challenges are especially pronounced in air pollution research, where the spatial resolution is often too broad to capture localized variability (44). Another important consideration is the mobility of participants during the study period, which can meaningfully impact analyses of perinatal outcomes, given variations in susceptibility throughout pregnancy and postpartum (24, 25, 31). Finally, while administrative health data help reduce recall bias, they often lack clinical detail and can inflate estimates. This particular challenge, combined with the absence of a gold standard diagnostic test for Kawasaki disease, increases the risk of misdiagnosis or outcome misclassification (45, 46).

Some of the results from the reviewed studies did not reach statistical significance, despite this overall trend of a positive association between increased particulate matter exposure and KD. Thus, as suggested by authors, future research should focus on validating this association across a range of study populations and geographical settings, and examine the biological and pathophysiological mechanisms involved (30). Addressing limitations in exposure assessment through individual-level exposure measurements, which are critical in air pollution research, may also strengthen the results (45, 46). Finally, as noted by Kwon et al., given the limitations of relying solely on clinical features for diagnosis, future studies may benefit from including objective inflammatory markers as intermediate indicators of Kawasaki disease (30).

## 5 Conclusion

This review explores the association between particulate matter (PM) exposure and the risk of developing and exacerbating Kawasaki disease (KD). Differentiation between aerodynamic sizes is particularly important. PM_10_, while linked to significant inflammatory responses, primarily affects the upper respiratory tract due to its limited ability to enter the bloodstream. Nevertheless, this inflammation, mediated by oxidative stress and cytokine production, may contribute to KD exacerbation, especially during mid-gestation with potential implications for maternal and child health. In contrast, PM_2.5_ penetrates the respiratory system and enters systemic circulation, triggering broader inflammatory and immune responses. Genetically predisposed children and individuals with preexisting inflammatory conditions, including metabolic syndromes, appear to be at increased risk due to heightened baseline inflammatory activity. Although not all reviewed studies reached statistical significance, the findings show a general positive association between exposure and disease and highlight the need for continued investigation into chronic air pollution exposure and its impact on KD. Future research should also examine the specific effects of these exposures on vulnerable populations to improve population health. In addition to these biological pathways, future research that integrates environmental, behavioral, and social modifiers into exposure assessment models will strengthen risk stratification, intervention targeting, and ultimately improve population health.

## Data Availability

The original contributions presented in the study are included in the article/Supplementary Material, further inquiries can be directed to the corresponding author.
