# Supplementary material for: Early life exposure to ambient particulate matter and Kawasaki disease: a systematic review and meta-analysis
Source: Front Cardiovasc Med. 2025 Aug 11;12:1611757. doi: 10.3389/fcvm.2025.1611757 (PMC12375594; doi:10.3389/fcvm.2025.1611757)
Supplement: Supplementary file 1 [file Datasheet1.pdf]

frontiers

Supplementary Material

S1. Search Terms

PUBMED

(kawasaki disease[MeSH Terms]) AND (air pollution[MeSH Terms])

"mucocutaneous lymph node syndrome"[MeSH Terms] AND "air pollution"[MeSH Terms]

(kawasaki disease[MeSH Terms]) AND (air pollutants[MeSH Terms])

"mucocutaneous lymph node syndrome"[MeSH Terms] AND "air pollutants"[MeSH Terms]

(kawasaki disease[MeSH Terms]) AND (particulate matter[MeSH Terms])

"mucocutaneous lymph node syndrome"[MeSH Terms] AND "particulate matter"[MeSH Terms]

EMBASE

('mucocutaneous lymph node syndrome'/exp OR 'mucocutaneous lymph node syndrome') AND ('air pollution'/exp OR 'air pollution')

('mucocutaneous lymph node syndrome'/exp OR 'mucocutaneous lymph node syndrome') AND ('air pollutant'/exp OR 'air pollutant')

('mucocutaneous lymph node syndrome'/exp OR 'mucocutaneous lymph node syndrome') AND ('particulate matter'/exp OR 'particulate matter')

S2. Search Methodology

A thorough search was conducted with a search engine and a database (PubMed, EMBASE), to ensure comprehensive coverage of relevant studies. The process initially captured 121 studies, which after screening using the Preferred Reporting Items for Systematic reviews and Meta-Analyses (PRISMA) protocol, yielded 11 studies meeting the inclusion criteria. The protocol for this systematic review was registered with the International Prospective Register of Systematic Reviews (PROSPERO) with registration number CRD42023468937.

Protocol	Justification
Exposures. Studies determining PM exposure, regardless of source, but with specific measurements of same were included.	The impact of PM_{10} and $PM_{2.5}$ on skin disease has been identified in primary studies with its effect associated with inflammatory and immunologic responses. To answer the research

	question, only studies focused on including the exposure to $PM_{2.5}$ were included in this study. Studies focused on other air pollutants without specific analysis of $PM_{2.5}$ exposure/outcome were excluded.
Types of outcome measures. Studies include measurable independent changes in risk, incidence or prevalence of Kawasaki disease. Studies included a reference group. Studies measuring only other forms of vascular disease were not included.	This outcome measure was specifically identified in the research question.
Types of studies. This systematic review considered all types of relevant studies using cohort, case-control, or cross-sectional designs and published in peer-reviewed journals that assess the association between Kawasaki disease and PM_{10} and $PM_{2.5}$ exposure. Reviews, commentaries, abstracts, and case reports were not included.	This approach ensured the systematic review incorporates only valid empirical evidence while minimizing bias and maintaining relevance to the research question.
Information sources. Despite a high number of duplicate studies and studies that did not meet the inclusion criteria in the initial search, the following search engines and databases were used to ensure all relevant papers were captured; PubMed, EMBASE.	This approach guarantees wider coverage of relevant journals.

S3. Risk of Bias Assessment – Quadas-2

	PATIENT SELECTION	INDEX TEST	REFERENCE STANDARD	FLOW AND TIMING
Buteau et al., 2020	Low	High	Low	Low
Jung et al., 2017	Low	High	Low	Low
Kim et al., 2024	Low	High	Low	Low
Kuo et al., 2022	High	High	Low	Low
Kwon et al., 2022	Low	High	Low	Low
Lin et al., 2017 6	Low	High	Low	Low
Oh et al., 2021	Low	High	Low	Low
Si et al., 2023	Low	High	Low	Low
Yoneda et al., 2024	Low	High	Low	Low
Yorifuji et al., 2018	Low	High	Low	Low
Zeft et al., 2016	Low	High	Low	Low

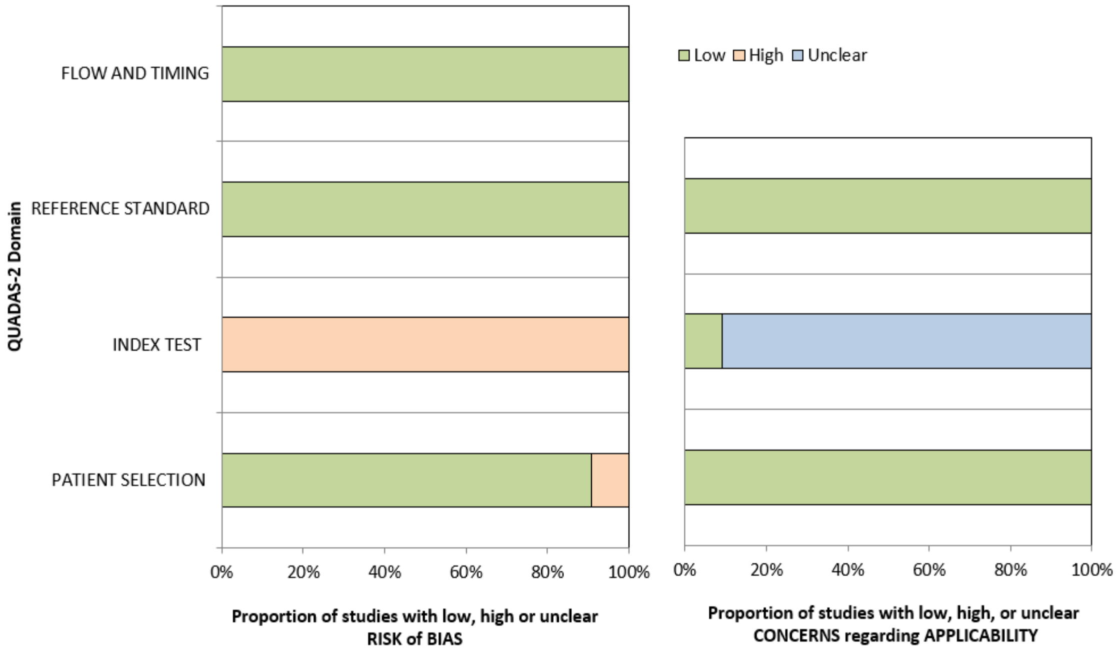

S4. Article selection after full-text review

Articles not selected

1. Corinaldesi E, Pavan V, Andreozzi L, et al (2020) Environmental factors and Kawasaki disease onset in Emilia-Romagna, Italy. Int J Environ Res Public Health 17:1529. https://doi.org/10.3390/ijerph17051529
2. Fujii F, Egami N, Inoue M, Koga H (2020) Weather condition, air pollutants, and epidemics as factors that potentially influence the development of Kawasaki disease. Sci Total Environ 741:140469. https://doi.org/10.1016/j.scitotenv.2020.140469
3. Jorquera H, Borzutzky A, Hoyos-Bachiloglu R, García A (2015) Association of Kawasaki disease with tropospheric winds in Central Chile: is wind-borne desert dust a risk factor? Environ Int 78:32–38. https://doi.org/10.1016/j.envint.2015.02.007
4. Konkel L (2017) Up in the air: Does ground-level ozone trigger Kawasaki disease? Environ Health Perspect 125:064003. https://doi.org/10.1289/EHP2092
5. Low T, McCrindle BW, Mueller B, et al (2021) Associations between the spatiotemporal distribution of Kawasaki disease and environmental factors: evidence supporting a multifactorial etiologic model. Sci Rep 11:14617. https://doi.org/10.1038/s41598-021-93089-9
6. Manlhiot C, Mueller B, O'Shea S, et al (2018) Environmental epidemiology of Kawasaki disease: Linking disease etiology, pathogenesis and global distribution. PLoS One 13:e0191087. https://doi.org/10.1371/journal.pone.0191087
7. Zhu Y, Chen R, Liu C, et al (2024) Short-term exposure to ozone may trigger the onset of Kawasaki disease: An individual-level, case-crossover study in East China. Chemosphere 349:140828. https://doi.org/10.1016/j.chemosphere.2023.140828

Articles not selected

1. Buteau S, Belkaibech S, Bilodeau-Bertrand M, et al (2020) Association between Kawasaki disease and prenatal exposure to ambient and industrial air pollution: A population-based cohort study. Environ Health Perspect 128:107006. https://doi.org/10.1289/EHP6920
2. Jung C-R, Chen W-T, Lin Y-T, Hwang B-F (2017) Ambient air pollutant exposures and hospitalization for Kawasaki disease in Taiwan: A case-crossover study (2000-2010). Environ Health Perspect 125:670–676. https://doi.org/10.1289/EHP137
3. Kim H, Jang H, Lee W, et al (2024) Association between long-term PM2.5 exposure and risk of Kawasaki disease in children: A nationwide longitudinal cohort study. Environmental Research 244:117823. https://doi.org/10.1016/j.envres.2023.117823
4. Kuo N-C, Lin C-H, Lin M-C (2022) Prenatal and early life exposure to air pollution and the incidence of Kawasaki disease. Sci Rep 12:3415. https://doi.org/10.1038/s41598-022-07081-y
5. Kwon D, Choe YJ, Kim S-Y, et al (2022) Ambient air pollution and Kawasaki disease in Korean children: A study of the National Health Insurance claim data. J Am Heart Assoc 11:e024092. https://doi.org/10.1161/JAHA.121.024092
6. Lin Z, Meng X, Chen R, et al (2017) Ambient air pollution, temperature and kawasaki disease in Shanghai, China. Chemosphere 186:817–822. https://doi.org/10.1016/j.chemosphere.2017.08.054
7. Oh J, Lee JH, Kim E, et al (2021) Is short-term exposure to PM2.5 relevant to childhood Kawasaki disease? Int J Environ Res Public Health 18:924. https://doi.org/10.3390/ijerph18030924
8. Si F, Zhou C, Yang Y, Huang L (2023) Study of the relationship between occurrence of Kawasaki disease and air pollution in Chengdu by parametric and semi-parametric models. Environ Sci Pollut Res Int 30:117706–117714. https://doi.org/10.1007/s11356-023-30533-5
9. Yoneda K, Shinjo D, Takahashi N, Fushimi K (2024) Spatiotemporal analysis of the association between Kawasaki disease incidence and PM2.5 exposure: a nationwide database study in Japan. BMJ Paediatr Open 8:. https://doi.org/10.1136/bmjpo-2024-002887
10. Yorifuji T, Tsukahara H, Kashima S, Doi H (2018) Intrauterine and early postnatal exposure to particulate air pollution and Kawasaki disease: A nationwide longitudinal survey in Japan. J Pediatr 193:147-154.e2. https://doi.org/10.1016/j.jpeds.2017.10.012
11. Zeft AS, Burns JC, Yeung RS, et al (2016) Kawasaki disease and exposure to fine particulate air pollution. J Pediatr 177:179-183.e1. https://doi.org/10.1016/j.jpeds.2016.06.061
